# Overall health impacts of a potential increase in cycle commuting in Stockholm, Sweden

**DOI:** 10.1177/14034948211010024

**Published:** 2021-05-12

**Authors:** Johan Nilsson Sommar, Christer Johansson, Boel Lövenheim, Peter Schantz, Anders Markstedt, Magnus Strömgren, Helena Stigson, Bertil Forsberg

**Affiliations:** 1 Department of Public Health and Clinical Medicine, Umeå University, Umeå, Sweden; 2 Department of Environmental Science, Stockholm University, Stockholm, Sweden; 3 Environment and Health Administration, SLB, Stockholm, Sweden; 4 The Research Unit for Movement, Health and Environment, The Swedish School of Sport and Health Sciences, GIH, Stockholm, Sweden; 5 WSP Civils, Stockholm, Sweden; 6 Department of Geography, Umeå University, Umeå, Sweden; 7 Folksam Research, Stockholm, Sweden; 8 Division of Insurance Medicine, Karolinska Institutet, Stockholm, Sweden

**Keywords:** Scenario, transport, bicycling, physical activity, air pollution, traffic injuries, health impact assessment, DALY

## Abstract

**Aims:**

To estimate the overall health impact of transferring commuting trips from car to bicycle.

**Methods:**

In this study registry information on the location of home and work for residents in Stockholm County was used to obtain the shortest travel route on a network of bicycle paths and roads. Current modes of travel to work were based on travel survey data. The relation between duration of cycling and distance cycled was established as a basis for selecting the number of individuals that normally would drive a car to work, but have a distance to work that they could bicycle within 30 minutes. The change in traffic flows was estimated by a transport model (LuTrans) and effects on road traffic injuries and fatalities were estimated by using national hospital injury data. Effects on air pollution concentrations were modelled using dispersion models.

**Results:**

Within the scenario, 111,000 commuters would shift from car to bicycle. On average the increased physical activity reduced the one-year mortality risk by 12% among the additional bicyclists. Including the number of years lost due to morbidity, the total number of disability adjusted life-years gained was 696. The amount of disability adjusted life-years gained in the general population due to reduced air pollution exposure was 471. The number of disability adjusted life-years lost by traffic injuries was 176. Also including air pollution effects among bicyclists, the net benefit was 939 disability adjusted life-years per year.

**Conclusions:**

Large health benefits were estimated by transferring commuting by car to bicycle.

## Introduction

An increasing proportion of the world population is living in growing cities with traffic congestion and air pollution concentrations exceeding decided limit values, requiring changes in within city transportation. Increasing the amount of bicycling in cities has the additional benefit of increasing the amount of physical activity. Both exposure to air pollution and lack of physical activity have been identified as the leading risk factors for disease [1]. The most recent update of long-term effects of air pollution performed by the World Health Organization (WHO), including studies published up until April 2012, found scientific evidence of associations with the incidence of cardiovascular diseases, diabetes mellitus, asthma and bronchitis, lung function, and mortality (total mortality and from respiratory and cardiovascular causes) (REVIHAAP, 2013) [2]. A systematic review has also been performed of prospective observational studies and intervention studies estimating the health benefits of active commuting (transport to work by walking and cycling) concluding that active transport through physical activity reduces the incidence of cardiovascular disease, diabetes mellitus, breast and colon cancer and mortality [3].

Using such risk functions the health impacts from increased bicycling have previously been reported, both within studies evaluating the benefits of public bicycle sharing systems [4–7] and studies evaluating hypothetical scenarios of increased bicycling (e.g. Rojas-Rueda et al. [5], de Hartog et al. [8] and Woodcock et al. [9]). No previous study has, however, used geocoded population data to assess the health impacts of increased bicycling. Using a road network including bicycle paths this also allowed for calculations of accumulated air pollution exposures for actual commuting trips among both bicyclists and motorists. This geographical information additionally provides the means to calculate the change in air pollution exposure within the general population with a high resolution.

High exposure to air pollutants occur both inside vehicles due to the proximity of air intakes to exhaust emissions from neighbouring vehicles as well as while walking or biking alongside roads [10]. Commuting by bicycle during rush hours along densely trafficked corridors may constitute a substantial fraction (20–30%) of the total daily exposure (e.g. Dons et al. [10] and Hanninen et al. [11]). Bicycling increases both intake and uptake of air pollutants as a result of an increased ventilation rate, depth of respiration and oral breathing. In a review of urban bicyclist’s exposures it was found that when bicycling between 12 and 24 km/h the minute ventilation was between 2 and 4.7 times higher compared to then driving a car [12].

Previous studies have assessed the negative aspects of bicycling by referring to fatalities or police reported injuries [4–9]. However, as most bicycle crashes are not reported to the police this study will use hospital reported data, covering a much higher rate of injuries from bicycle crashes, to estimate the effect on injuries and fatalities from traffic crashes when shifting the mode of transport from car to bicycle by using hospital reported data.

This study aims to estimate, using registry data on coordinates of home and work place and a network of bicycle paths and roads available for bicycling, the overall health impacts in a scenario of increased bicycling including: (a) the benefit of increased physical activity; (b) the risk of increased air pollution exposure for the individuals that change their mode of transport from car to bicycle; (c) the benefit of reduced air pollution exposure among current bicycle commuters; (d) the benefit of reduced air pollution exposure among the general population; and (e) the increased injury risk comparing bicycling with driving a car.

## Methods

### Current modes of commuting

The proportion currently travelling to work with each mode of transport (walking, bicycling, public transport, passenger of a car and driver of a car) was estimated based on travel survey data obtained for the study population [13]. The survey data included trips and modes of travel for a specific day. These proportions were estimated by aggregating individual survey responses to small statistical areas within Stockholm County. The size of these areas is determined by the population density, but also taking into consideration natural geographical divisions between areas.

Using data on traffic flows on roads together with estimated proportions of individuals utilising different modes of travel a traffic model for Stockholm County has been constructed (the LuTrans transport model) [14]. Regularly calibrated based on repeated measures of traffic flows the LuTrans transport travel output is modelled using a logit model of: (a) travel survey data allocating individual travels to different modes of transport and (b) traffic counts to allocate car trips to different roads. The resulting transport system output is estimated by the traffic flow on each road link, in which a link is defined as the connection between two major intersections within the road network.

Based on this model all inhabitants with a home and work address within Stockholm County was allocated to a current mode of transport [15]. This allocation also considered individual data on car ownership. Individual information on age, gender, car ownership and home and work addresses was obtained from the ASTRID database [16].

### Constructing the car to bicycle scenario

Individual data in terms of age, sex and car ownership from the ASTRID database was also used to identify the individuals that have the potential to bicycle to their workplace within 30 minutes. The database also contained individual coordinates for the home and work address. Based on these coordinates the shortest path along a network of possible roads and bicycle paths was determined. Data on age and gender contained in the ASTRID database were used to predict the expected individual average bicycle speed. If the individual was estimated to have the potential to bicycle to work within 30 minutes, and the individual was previously allocated as travelling to work by driving a car, the individual will in the scenario switch to commute by bicycle.

Expected bicycling speeds were derived based on empirical distance and bicycling time relationships within a sample of 455 existing male and female bicycle commuters within the population of Greater Stockholm, Sweden. The recruitment of study participants was performed through newspaper advertisements, and the characteristics of study participants have previously been described in Schantz (2017) [17]. Participants marked their own normal bicycle commuting route to work on a map, and the bicycle distance was measured using a criterion method [18]. As described by Schantz [17] the participants provided self-reported measures of their bicycling time to and from work, without any errands on the way. As these estimated time–distance relationships may not be representative for the general population, these were scaled down by age and gender-specific relative differences in maximum oxygen uptake between current bicycle commuters and a sample from the general population. The downscaling of expected bicycling speeds to represent the general population has been described in detail by Schantz et al. (2018) [19], in which resulting bicycle speeds were given by

speed (km/h) = 0.719 * (34.8 + 0.31 * age) among men

and

speed (km/h) = 0.763 * (25.9 + 0.21 * age) among women,

and where age was measured in years, and 0.719 and 0.763 represent the cycle commuter to the general population effect among men and women, respectively.

The LuTrans traffic model was also used to predict traffic flows in the car to bicycle scenario where car trips were transferred to bicycle. By constructing a demand matrix for the amount of traffic on road sections the selected commuting route for each individual car trip was obtained, and as the demand within the road system decreases due to the reduced number of cars a new traffic flow was estimated in which remaining traffic may choose a different route. Each road is defined by short road links and the result of such a change will be at the road link level which thereafter will be used to calculate vehicle emissions and traffic air pollution concentrations on these links.

To calculate air pollution exposure among bicycle commuters it was necessary to know where the bicycle network is separated from the road network. For this purpose the bicycle network was imported from digital data provided by the national road database (NVDB) together with the local road databases (LVDBs).

#### Estimating the amount of physical activity

Using the individual’s shortest path bicycling distance between home and work and the individual’s expected bicycle speed based on age and gender, the bicycling time was estimated. The bicycling intensity was assumed to be 6.8 metabolic equivalents (METs) based on measurements for bicycle commuting [20]. Thereafter the yearly amount (MET-hours/week) of physical activity was estimated assuming four round trips a week 45 weeks a year. The motive for this volume of physical activity is that four round trips mirrors the median behavioural pattern during the cycling season [21], and 45 weeks of cycle commuting per year represent an all-year round cycling behaviour (leaving out the vacations and holidays).

#### Vehicle emissions

Approximately 40,000 road links and an annual traffic volume of 12,000 million vehicle km within Greater Stockholm was used as the basis for the emission inventory [22]. The road traffic emission of nitrogen oxides (NOx), particulate matter (PM)-exhaust and black carbon (BC) were estimated using emission factors (emission per km driven) in which vehicles were grouped into passenger cars (petrol and diesel), light commercial vehicles, heavy goods vehicles and buses. Emission factors for NOx and PM-exhaust for different vehicle types, speeds and driving conditions were determined using the Handbook Emission Factors for Road Transport (HBEFA) 3.2 [23]. The emission factors for BC were based on estimates from the Transphorm project [24].

#### Dispersion modelling of air pollutants

The air pollution concentrations were modelled using the same meteorological conditions within both the current traffic situation and the mode shift scenario. A wind tunnel model and a Gaussian air quality dispersion model, both part of the Airviro Air Quality Management System (http://airviro.com), were used to model local road traffic emissions. This system has been in operation in Stockholm for more than 20 years and has been used for modelling air pollution concentrations within both epidemiological and health assessment studies [25–27].

The meteorological data used were based on a climatology created from 15 years of meteorological measurements (15 minute averages) in a 50 m high mast located in the southern part of Stockholm. The resulting climatology was calculated as the frequency of occurrences of events that together build a distribution of weather conditions (for further details see Johansson et al. [26]).

Dispersion calculations were performed on a 25 m square resolution including the average diurnal variation in concentrations along different roads depending on the traffic volume, composition and atmospheric dispersion. The effects of buildings on the dispersion were considered using a street canyon model part of the Airviro system. The concentrations along streets were used to estimate the exposure dose for the individuals bicycling instead of driving a car.

To estimate the air pollution exposure for bicyclists and motorists the results from the dispersion modelling were adjusted to represent weekdays in April to October (excluding July) during morning rush hours between 7 a.m. and 9 a.m. The concentration for each road link was estimated as the weighted mean of intersecting 25 m square grids within a GIS program.

The accumulated air pollution concentration along the routes taken as a bicyclist and as a motorist, respectively, was calculated as the sum of the link concentration (C_i_) times the time spent on that link (t_i_):

$$\sum_{i=1}^{n}C_{i}t_{i}$$

where *i* = (1, 2, . . ., *n*) is the link sequence between home and work.

Doses of NOx, particles with a diameter of 10 μm or less (PM10), particles with a diameter of less than 2.5 µm (PM2.5), PM due to vehicle exhaust emissions (PM-exhaust) and BC were calculated assuming four round trips a week 45 weeks a year. Time not spent commuting was assumed to be spent at home. Population weighted exposures at home addresses were retrieved from Johansson et al. (2017) [28]. The change in ambient concentrations, population exposure and the impact on mortality among citizens have been reported without any analysis of in-traffic exposure [28].

#### Estimation of air pollution doses among bicyclists and motorists

Within the epidemiological studies on the effects of long-term air pollution exposure in adults it is the outdoor concentration that is measured or modelled. The study participants can, however, on average be assumed to have spent 90–95% of their time indoors. For fine particles and nitrogen dioxides (NO_2_) judged to be important for health effects the proportion of the outdoor concentration that infiltrates buildings has typically been found to be approximately 40–60% [29], [30]. Also the infiltration into car cabins has on average been reported to be of the same magnitude [31], [32], but depends on a number of factors including the vehicle ventilation, filter efficiency and particle size distribution [33], [34]. We have assumed that air pollutant concentrations inside the car cabin were 50% of the outdoor concentration. This means that: (a) the resulting change in actual air pollution exposure therefore was calculated to be half of the calculated change in outdoor concentration; and (b) that the relative risk increment was assumed to be half of that reported in the epidemiological studies.

Based on results by Daigle et al. (2003) [35] we also assumed the minute ventilation to be 3.3 times higher for bicyclists compared with at rest indoors or sitting in a car, and that uptake is increased by 31% resulting in an increased dose of 430%.

Based on these assumptions about bicyclists’ and motorists’ exposures we calculated: (a) the contribution to the yearly mean exposure as a result of the increased air pollution dose for the individuals that previously drove a car to work but instead start to bicycle; and (b) the reduction of the yearly mean exposure among current bicycle commuters. As the travel time between home and work may differ as a bicyclist compared to as a motorist, the mean exposure at the home address was used for the time not spent commuting. The details on these calculations have previously been published [36].

#### Health impact calculations

##### Physical activity

Using the risk reduction (RR) function for all-cause mortality in relation to bicycling used in the health economic assessment tool (HEAT) [37], the reduced number of yearly pre-term deaths with increased physical activity was calculated. The RR implemented was 10% (95% confidence interval (CI) 6–13%) for the standardised amount of physical activity corresponding to 11.25 MET-hours/week. This RR is the result of a meta-analysis of seven studies. Six of these studies were based on populations within western Europe (four from Denmark and one study from UK and Germany, respectively). The bicycling assessed was predominantly commuting. All but one study reported a reduced risk of all-cause mortality with bicycling. The meta-analysis was based on 187,000 individuals observed during in total 2.1 million person-years. The mean age during the follow-up was 56.6 years of age, ranging between 20 and 93 years.

The implemented RR for morbidity outcomes associated with commuting and leisure time physical activity was based on a review with meta-analyses of prospective cohort studies published between 1990 and 2015 [38]. Included studies reported the amount of physical activity as MET-hours/week or day, or intensity and duration. Among studies on commuting physical activity reporting only the duration of activity the physical activity intensity was assumed to be 6.8 and 4 METs for bicycling and walking, respectively. The implemented RR estimates for each 11.25 MET-hours/week of physical activity were 18% (95% CI 1–33%) for myocardial infarction, 23% (95% CI 9–35%) for stroke, 27% (95% CI 14–38%) for heart failure (HF), 22% (95% CI 4–37%) for type 2 diabetes, 7% (95% CI 2–12%) for breast cancer and 15% (95% CI 6–22%) for colon cancer.

##### Air pollution

The epidemiological studies with a finer spatial resolution which can capture the gradients in exposure to local traffic pollutants indicate an important effect of local traffic emissions, resulting in high relative risks [39], [40]. Of particular interest is a Norwegian study of 16,000 men from Oslo, of whom 25% died during the follow-up, which used modelled NOx in the residential area as the exposure indicator [41]. This cohort, with people of between 40 and 49 years of age at the start of the study, was followed from 1972/1973 to 1998. NOx was estimated in a model with 1000 m grids, and a street contribution was added for the largest streets. When the median concentration of NOx for 1974–1978 was used, the relative risk for total non-violent mortality was 1.08 per 10 µg/m^3^ (95% CI 1.06–1.11).

Also for BC, cohort studies have provided sufficient evidence of associations between all-cause and cardiopulmonary mortality with long-term average BC exposure. Studies of short-term health effects show that the associations with BC are more robust than those with PM2.5 or PM10, suggesting that BC is a better indicator of harmful particulate substances from combustion sources (especially traffic) than undifferentiated PM mass. For BC we used the pooled estimate for premature mortality associated with long-term exposure to elemental carbon of 6% per 1 μg/m^3^ (95% CI 5–7%) as reported in a review by Hoek et al. (2013) [42].

Epidemiological evidence also exists for associations between air pollution and the incidence of chronic bronchitis, myocardial infarction, stroke, type 2 diabetes and lung cancer. A meta-analysis of American and European studies performed within HRAPIE found that the risk of chronic bronchitis increased by 11.7% for each 10 μg/m^3^ increase in PM10 [43]. Within the European ESCAPE project, a meta-analysis of included cohorts showed a 33% risk increase for the incidence of stroke per 5 μg/m^3^ increase of particles with a diameter of 2.5 μm (PM2.5) or less [44]. Also the incidence of lung cancer and coronary events was analysed within ESCAPE, reporting a 22% [45] and 12% [46] increased risk per 10 μg/m^3^ PM10, respectively. For diabetes a study from Germany found a 5% increased risk per 10 μg/m^3^ increase of PM10 [47]. Implemented exposure–response functions are presented in Table I.

**Table I.** Implemented relative risk estimates and disability weights used to calculate expected health impacts and disease burden.

Exposure		Outcome	Disability weight	Relative risk	Increment (μg/m^3^)	Original study	Age group (years)
Exhaust	NOx	Mortality		1.08 (1.06–1.11)	10	Nafstad et al., 2004 [41]	All ages
	BC	Mortality		1.06 (1.05–1.07)	1	Hoek et al., 2013 ^42^	>30
	PM2.5	Stroke	Long term 0.171 Short term 0.92	1.33 (1.01–1.77)	5	Meta-analysis by Staffogia et al., 2014) [44]	>30
Traffic related air pollution	PM10	Lung cancer	0.15	1.22 (1.03–1.45)	10	Meta-analysis by Raaschou-Nielsen et al., 2013 [45]	>30
	PM10	Myocardial infarction	0.395	1.12 (1.01–1.25)	10	Meta-analysis by Cesaroni et al., 2014 [46]	>30
	PM10	Type 2 diabetes	0.033	1.05 (1.00–1.10)	10	Weinmayer et al., 2015 [47]	>30
	PM10	Chronic brochitis	0.043	1.12 (1.04–1.19)	10	HRAPIE (WHO, 2013) [43]	>30

BC: black carbon; NOx: nitrogen oxides; PM: particulate matter.

##### Injuries

A national registry database (STRADA) containing information about traffic fatalities and injuries reported by police or emergency care hospitals was used to estimate the impact on road traffic injuries and fatalities (Swedish Traffic Accident Data Acquisition; Swedish Transport Agency, 2016). Only injuries occurring among individuals aged 18–65 years old were included to make the data representative of the individuals that in the scenario shifted mode of transport from car to bicycle. Swedish national travel survey data were used to obtain estimates of mode-specific traffic mileages. Thereafter the injury risk per distance travelled was calculated for each mode. These risks were used to estimate the expected impact on injuries due to a mode shift from car to bicycle based on the change in number of km travelled by car and bicycle. The implemented risks were calculated from currently available data and were, per km, assumed to remain the same also in the mode shift scenario. The impact on fatalities and injuries for the current scenario has previously been published [48], including a detailed description of the traffic injury modelling.

##### Measure of impact

The estimated health impacts due to increased physical activity and changes in air pollution exposure were reported as the expected impact on the number of pre-term deaths and incident cases of included diseases. Resulting impacts on the disease burden were calculated as the sum of expected years of life lost and expected years lost to disability. The impact of injuries was calculated in the same manner based on expected years of life lost due to fatalities and injury-specific expected years lost to disability obtained from Tainio et al. (2014) [49]. These disability adjusted life-years (DALYs) were calculated for each area of impact (physical activity, air pollution and injuries) and were used to calculate the net benefit for the disease burden in the population of Stockholm County. The implemented disability weights, presented in Table I, were based on treated patients and were obtained from the WHO Global Burden of Disease 2004 update [50].

Expected disease durations were calculated using age and gender-specific registry data on yearly mortality after the first incidence of disease [51]. For myocardial infarction the duration of disability was assumed to be at most one year. The duration of disability for the other included diseases was calculated as the expected life length after the first disease incidence. For stroke the expected life length after the first year of survival was obtained from a study using the Swedish stroke register [52], with a linear (straight line) extrapolation after 3 years survival. The mortality after heart failure was assumed to be on average three times as high as in the general population, whereas no increased mortality was assumed after incident diabetes.

Age and gender-specific baseline mortality and disease incidences were obtained from registry data for Stockholm County [51]. The incidence of type 2 diabetes was, however, obtained from a Swedish national register study on pharmacologically treated diabetes [53] and the incidence of chronic bronchitis from a questionnaire study within a county in western Sweden [54]. The definition of chronic bronchitis was a chronic productive cough for at least 3 months per year during two consecutive years. The baseline mortality in year 2013 for individuals older than 30 years of age within Stockholm County was 1124 per 100,000. Life-table calculations for Stockholm County mortality data were used to calculate expected remaining life-years based on age and gender.

## Results

### Travel mode distributions

Residential and work address data together with registry data on employment identified 923,970 individuals that had their home and work address within Stockholm County. Their mean age was 42 years and 49% were women. Currently, 6% were estimated to commute to work by bicycle, 38% drove a car, 4% as passenger of a car, 38% by public transport and 14% walked (Table II). Within the mode shift scenario an additional 111,487 individuals were allocated to commute by bicycle. In this scenario 18% would travel by bicycle and 26% would drive a car.

**Table II.** Frequencies and proportions of the individuals utilising different types of transport.

Mode of transport	Current situation		Mode-shift scenario		Difference and corresponding proportion	
	No. of individuals	Proportion	No. of individuals	Proportion	No. of individuals	Proportion
Bicycling	53,206	6%	164,693	18%	111,487	210%
Walking	130,441	14%	130,441	14%	0	0%
Public transport	352,412	38%	352,412	38%	0	0%
Car (driver)	352,614	38%	241,127	26%	–111,487	–32%
Car (passenger)	35,297	4%	35,297	4%	0	0%

### Physical activity

#### Estimated amounts of physical activity

Median bicycle distance to work was 3.5 km among current bicyclists compared to 3.3 km among the additional bicyclists in the scenario. The mean age among these additional bicyclists was 42 years and 48% were women. Based on age and gender-specific modelled bicycle speeds the average individual cycling time between home and work was calculated. Average bicycle times among current bicyclists were estimated to be 22 minutes and among the additional bicyclists in the mode shift scenario 15 minutes. The average amount of physical activity performed by these additional bicyclists was 13 MET-hours/week, with individual amounts ranging up to 27 MET-hours/week.

#### Health impacts

Due to the increase of physical activity among the individuals that were transferred from driving a car instead to commute by bicycle the risk of yearly pre-term mortality decreased on average by 12%. Using age and gender-specific mortality data for Stockholm County the resulting impact on mortality would be 16.2 yearly prevented pre-term deaths. These deaths were estimated to correspond to 469 yearly gained life-years. The greatest impact on morbidity was found for type 2 diabetes in which the incidence was reduced by 127 cases yearly corresponding to 94 years of life lost to disability (Table III). In total, 33% of the resulting reduction in DALYs was achieved due to a reduced morbidity among the additional bicyclists, whereas the remaining 67% was due to the reduction in pre-term mortality.

**Table III.** Estimates of the disease incidence, mortality and burden of disease within the current situation, the mode shift scenario and resulting health impact.

Physical activity				
	Exposure	Current situation	Mode shift scenario	Health impact
Morbidity: YLD (no. of cases)				
Breast cancer	Bicycling	186 (73)	170 (67)	–15.6 (–6.1)
Myocardial infarction	Bicycling	24 (77)	19 (61)	–4.8 (–15.6)
Stroke	Bicycling	197 (69)	140 (49)	–57.4 (–20.0)
Diabetes (type 2)	Bicycling	397 (342)	295 (255)	–102.1 (–87.3)
Heart failure	Bicycling	120 (37)	83 (26)	–36.8 (–11.1)
Colon cancer	Bicycling	60 (16)	50 (13)	–10.5 (–2.8)
Mortality: YLL (no. of cases)	Bicycling	4091 (143)	3622 (127)	–469 (–16.2)
DALYs		5074	4379	–695
Change in the population’s air pollution exposure				
	Exposure	Current situation	Mode shift scenario	Health impact
Morbidity: YLD (no. of cases)				
Stroke	PM2.5	5600 (3585)	5583 (3573)	–16.9 (–11.9)
Myocardial infarction	PM10	344 (1564)	344 (1563)	–0.3 (–1.4)
Diabetes (type 2)	PM10	5641 (6104)	5639 (6102)	–1.9 (–2.2)
Lung cancer	PM10	879 (708)	878 (707)	–1.4 (–1.1)
Asthma	PM10	2517 (1527)	2515 (1526)	–1.6 (–1.1)
Mortality: YLL (no. of cases)	NOx	138798 (12704)	138349 (12641)	–449 (–63)
DALYs		150074	149603	–471.1
Change in air pollution exposure among the additional bicyclists				
	Exposure	Car use	Bicycling	Health impact
Morbidity: YLD (no. of cases)				
Stroke	PM2.5	183 (63)	187 (64)	3.20 (1.14)
Myocardial infarction	PM10	21 (69)	21 (70)	0.26 (0.88)
Diabetes (type 2)	PM10	360 (314)	362 (315)	1.92 (1.73)
Lung cancer	PM10	18 (15)	19 (16)	0.42 (0.35)
Asthma	PM10	157 (87)	158 (88)	1.60 (0.97)
Mortality: YLL (no. of cases)	NOx	3672 (133)	3720 (135)	47.9 (1.69)
DALYs		4411	4466	55.3
Change in air pollution exposure among previous bicyclists				
	Exposure	Current situation	Mode shift scenario	Health impact
Morbidity: YLD (no. of cases)				
Stroke	PM2.5	93 (31)	93 (31)	–0.08 (–0.03)
Myocardial infarction	PM10	10 (32)	10 (32)	0.00 (–0.01)
Diabetes (type 2)	PM10	164 (142)	164 (142)	–0.02 (–0.02)
Lung cancer	PM10	9 (8)	9 (8)	–0.01 (0.00)
Asthma	PM10	79 (43)	79 (43)	–0.02 (–0.01)
Mortality: YLL (no. of cases)	NOx	1735 (65)	1732 (64)	–2.63 (–0.10)
DALYs		2090	2087	–2.8
**Traffic injuries**				
DALYs				176
**Total change in DALYs**				–938.8

Calculations are presented for (a) the increase in physical activity among the individuals that changed mode of transport from car to bicycle; (b) the change in air pollution exposure within the general population; (c) the change in air pollution exposure among the additional bicyclists; (d) the change in air pollution exposure among previous bicyclists; and (e) the change in traffic injuries.

Impacts were presented as cases, years lost to disability (YLD), years of life lost (YLL) and disability adjusted life-years (DALYs).

### Air pollution

#### Concentrations within the general population

Among the general population within Stockholm County the current average concentrations of NOx, BC, PM2.5 and PM10 at individuals’ home addresses were 7.91, 0.65, 5.44 and 11.4 μg/m^3^, respectively. In the mode shift scenario these concentrations were calculated to be reduced by 0.33, 0.023, 0.057 and 0.07 μg/m^3^, respectively. Relative to current concentrations these reductions corresponded to 4.2%, 3.5%, 1.0% and 0.6%.

#### Concentrations among bicyclists and drivers of a car

Time-weighted average NOx concentrations along bicycle paths and roads available for bicycling were among current bicyclists 25.8 μg/m^3^ and in the mode shift scenario 24.2 μg/m^3^. For the individuals that changed their mode of transport from car to bicycle time-weighted NOx concentrations were 15.0 μg/m^3^ as motorists in the current scenario and 21.1 μg/m^3^ as bicyclists in the mode shift scenario. For BC, corresponding concentrations among current bicyclists were 1.14 μg/m^3^ and 1.08 μg/m^3^, and among the additional bicyclists 0.75 μg/m^3^ and 0.96 μg/m^3^. The time-weighted PM10 concentration along paths among current bicyclists was reduced from 14.4 μg/m^3^ to 14.1 μg/m^3^ (for PM-exhaust 0.44 to 0.41, and PM2.5 4.78 to 4.76) and among the additional bicyclists increased from 12.2 μg/m^3^ to 13.5 μg/m^3^ (for PM-exhaust 0.23 to 0.35, and PM2.5 6.71 to 7.75) comparing concentrations along streets travelled as the driver of a car with concentrations along bicycle paths as a bicyclist in the mode shift scenario.

#### Air pollution dose calculations

Although time-weighted average concentrations were found to be higher as a bicyclist compared with car drivers, considering the increased intake and uptake among bicyclists and the lower concentration inside the car compared with outside as well as the on average longer travelling time as a bicyclist, the air pollution doses were found to be several times higher among bicyclists compared with as the driver of a car. On average, the contribution to the yearly mean NOx dose increased from 0.08 μg/m^3^ to 1.03 μg/m^3^ among the individuals that changed their mode of transport from car to bicycle. Among current bicyclists the contribution to the yearly mean decreased from 1.71 μg/m^3^ to 1.61 μg/m^3^. For BC, corresponding contributions to the yearly mean changed from 0.08 μg/m^3^ to 0.07 μg/m^3^ among current bicyclists and 0.004 μg/m^3^ to 0.05 μg/m^3^ among the individuals that changed their mode of transport to bicycle. PM10 dose contributions changed from 0.97 μg/m^3^ to 0.96 μg/m^3^ (for PM-exhaust 0.030 to 0.028, and PM2.5 0.58 to 0.56) among current bicyclists and 0.07 μg/m^3^ to 0.66 μg/m^3^ (for PM-exhaust 0.0013 to 0.017, and PM2.5 0.04 to 0.38) among the additional bicyclists in the car to bike scenario.

#### Health impacts

The 0.33 μg/m^3^ decreased NOx concentration within the general population was expected to reduce the yearly mortality by 63 preterm deaths corresponding to 449 yearly gained life-years. The impact on mortality related to the 0.023 μg/m^3^ reduced BC concentration would be 32 preterm deaths corresponding to 185 yearly gained life years. However, both NOx and BC cannot be used without potential double counting due to their correlation and unadjusted relative risk estimates. The impact on morbidity related to the reduction in PM10 concentrations was found to be limited, with the largest impact on stroke for which the incidence in the general population would be reduced by 12 cases per year (Table III).

Based on changes in NOx doses the yearly mortality decreased by 0.10 deaths corresponding to 2.63 gained life-years among current bicyclists, and increased by 1.69 yearly preterm deaths corresponding to 47.9 years of life lost among the individuals that changed their mode of transport to bicycle. The impacts based on BC concentrations were found to be lower, 0.02 fewer preterm deaths and 0.59 gained life-years among current bicyclists and 0.48 more preterm deaths corresponding to 13.4 years of life lost among the individuals that changed their mode of transport to bicycle.

### Net impact

In total the impact from the increase in physical activity among the individuals that changed their mode of commuting from car to bicycle was 696 DALYs. The second largest impact was found as a result of the reduced air pollution exposure within the general population, resulting in 471 DALYs. Among the individuals that changed their mode of commuting to bicycle the risk for traffic accident injuries was expected to increase and fatalities and injuries were in total calculated to cause 176 DALYs each year. The increase in air pollution dose among the additional bicyclists was calculated to increase morbidity and mortality corresponding to 55 DALYs, and a small impact was also calculated for current bicyclists corresponding to a 3 DALYs decrease. The total health impact of the scenario was estimated to be 939 DALYs. When calculating the net impact including mortality in relation to only BC the total health benefit was reduced to 730 DALYs.

## Discussion

To the best of our knowledge this is the first health impact assessment (HIA) of increased bicycling based on registry data. Using information on home and work addresses the health impacts were calculated for actual commuting trips. Registry data also allowed for the implementation of individual bicycling speeds based on age and gender using empirical time–distance relationships within the study population. These individual bicycling speeds, coordinates for home and work addresses together with a network of bicycle paths and roads, and dispersion modelled air pollution concentrations facilitated calculations of accumulated air pollution exposures as the driver of a car and as a bicyclist. The health impact findings for a mode shift from car to bicycle showed that the largest reduction in the number of pre-term deaths would be due to decreased air pollution exposure within the general population, whereas the number of gained life-years was about the same as the impact of the increase in physical activity. As the largest impact on the incidence of disease was found in relation to increased physical activity, the largest total impact was obtained due to increased amounts of physical activity. The greatest impact on the disease incidence and years of life lost due to disease was found for type 2 diabetes. As also found within previous HIAs on increased bicycling the health impact due to reduced disease incidence was small in terms of DALYs compared to the impact on mortality [55]. The increased air pollution exposure among the individuals that changed their mode of transport to bicycling contributed to a comparatively small disease burden; however, the HIA calculated that there was an additional potential for health benefits from bicycling by reducing within city traffic-related air pollution concentrations.

Within previous HIAs on increased bicycling the largest impact has been found due to increased physical activity (e.g. Rojas-Rueda et al. [5], de Hartog et al. [8] and Woodcock et al. [9]), except for one study evaluating a transport scenario in which drivers of a car to a large extent were also transferred to the public transport system or to be the passenger of a car [56]. Such a mode shift from car to public transport resulted in reduced risks for traffic accident injuries, corresponding to about half of the total health impact. The study by Dhondt et al. (2013) [56] is also one of the two previous studies that estimated large health impacts related to a reduced air pollution exposure within the general population. The study assessed the air pollution impact considering changes in annual elemental carbon concentrations using a meta-analysis estimate related to all-cause mortality [57]. The other study estimating a large air pollution impact on the general population assessed the impact by eliminating all automobile round trips less than 8 km within several metropolitan US areas. Half of these trips were assumed to be transferred to bicycling, and population air pollution impacts were assessed based on changes in annual mean concentrations of PM2.5 and ozone. The other studies have estimated health impacts in relation to calculated changes in PM2.5 concentrations.

As presented in a review of air pollution as a risk factor in HIAs of a travel mode shift towards bicycling, studies calculating health impacts based on exposure–response functions for NO_2_, BC and ozone have found comparatively larger health impacts related to air pollution exposure compared with studies evaluating health impacts related to PM2.5 [58]. This is to a large extent due to the relative risk function used for calculating health impacts in relation to changes in annual PM2.5 concentrations. The most commonly used exposure–response function for PM2.5 is based on studies comparing background concentrations of particles between cities. These studies have been based on urban background monitoring, and therefore to a large extent reflect secondary (non-local) PM (e.g. Hoek et al. [42]). We suggest that NOx, NO_2_ and BC are better indicators of health impacts. All three are indicators of adverse health effects associated with vehicle exhaust emissions. In particular, NOx and BC are highly correlated with other toxic constituents in vehicle exhaust and therefore it is difficult to judge independent effects of either indicator. Both NOx and BC have been shown to be associated with increased premature mortality [41], [57], [59]. Epidemiological studies with a finer spatial resolution which can capture the gradients in exposure to local traffic pollutants indicate an important effect of local traffic emissions, resulting in high relative risks [39], [40].

Previous assessments of the health impact of air pollution exposure during bicycling have also been based on concentrations of PM2.5 [5], [60–67], one study, however, also estimated the health impacts in relation to BC [8]. The scenario considered by de Hartog et al. (2010) [8] transferred 500,000 individuals’ short car trips to bicycle. Based on resulting changes in PM2.5 and black smoke concentrations the risk increase for pre-term mortality was found to be five times as high when assessed in relation to BC.

According to a review of HIA studies of increased bicycling between 12% and 99% of the total impact on health was found to be attributed to increased physical activity [55]. A linear dose–response (straight line) between the RR for yearly mortality and the amount of physical activity but with a maximum RR of 50% was used within most HIA studies [6], [9], [56], [65]. Assessments of the shape of the association have, however, found that it is rather non-linear [68], [69]. Woodcock et al. (2011) [9] found that when comparing the Integrated Transport and Health Impact Modelling Tool (ITHIM) model with a square root dose–response between physical activity and all-cause mortality the percentage reduction in pre-term deaths was about the same as when using the straight line fit based on the meta-analyses of bicycling studies by Kelly et al. (2014) [68], the RR was between 0.82 and 1.02 times as high for the different scenarios when applying the straight line fit. The comparison was made within the age group 20–64 years of age. Implementing a non-linear function, however, adds uncertainty due to the necessity to include data about individuals’ amounts of other types of physical activity.

Assessing the health impact of increased bicycling it is also necessary to make assumptions about how this would affect the individuals’ other physical activity domains. Increased physical activity through active commuting by bicycle may replace other types of physical activity, but it is also possible that increased active commuting increases physical activity in general. Existing longitudinal epidemiological findings suggest that walking and bicycling on average add to the total amount of physical activity, without replacing physical activity in other domains [70], [71].

The amount of traffic accident fatalities and injuries has for a mode shift to bicycle in previous HIAs generally been found to increase. Some studies have, however, found that this burden of traffic injuries would be expected to decrease due to the fact that the scenario also implied a mode shift to public transport that has a lower injury risk compared to driving a car. The negative effect on injuries might have been underestimated in previous studies because mainly police reported crash data [54] and fatality data [12] have been used. Very few bicycle crashes are known by the police and therefore it is essential to use hospital data to make realistic estimations. In the current study we were able to use hospital reported data on injuries that are representative for Sweden (in 2014 all but one emergency care hospital in Sweden was included in STRADA) making it possible to more accurately estimate the impact from several types of injuries.

In summary, the interpretation of the finding of a comparatively large impact due to reduced air pollution exposure within the general population was obtained by a mode shift of 110,000 current car commuters to bicycle, and by implementing relative risks from studies that assess air pollution associations considering local sources. The net impact on traffic injuries caused by the mode shift was obtained by, beside fatalities, also include injuries from hospital records, and by calculating the injury risk difference, for instance, by subtracting neck injuries among motorists from rear-end collisions.

HIAs are generally limited by the availability of data, requiring assumptions to be made. Compared to previous studies the study benefitted from the use of individual registry data for the entire study population, including home and work address coordinates, which made it possible to perform a HIA of actual commuting trips. However, individual data on the mode of transport were not available and therefore imputation of mode of transport within areas was performed according to travel survey information and information on car ownership. Using a network of bicycle paths and roads available for bicycling we were also able to extract the shortest bicycling path between home and work. The bike paths contain traffic stops; however, bicyclists were assumed to maintain a constant speed. The concentrations of air pollutants may be higher in these traffic intersections than elsewhere on the bicycle route and therefore air pollution dose estimates may be somewhat underestimated. Also, even though drivers of a car may choose to take a different route if there is less traffic congestion within the scenario with increased bicycling, we were in the scenario not able to incorporate such congestion when estimating the cumulative air pollution exposure among drivers. Instead these accumulated dose estimates among drivers were estimated assuming flowing traffic. In addition, as air pollution components are location and source specific, using air pollution risk estimates from elsewhere introduces uncertainties regarding relative risks and resulting impacts [72]. Regarding air pollution dose estimation, a recent measurement study on ventilation and intensities among cycle commuters found that these may be higher than previously assumed within HIAs [73].

Empirical data on the time–distance relationship among current bicycle commuters within the study population was used to obtain gender and age-specific expected bicycle speeds to assess the individual capacity to bicycle the distance between home and work within 30 minutes. The same bicycling intensity was, however, used due to the lack of studies on age, gender and bicycling speed intensities for active commuting. Using average bicycling intensities affects the estimate of physical activity for the individual; however, not for the average amount of physical activity among all additional bicyclists in the scenario, and therefore would not bias the total impact on health. The upper limit for the bicycling time to work was arbitrarily chosen to be 30 minutes but aimed to create a reasonably realistic scenario in terms of travelling time. The average bicycling time among current bicycle commuters in the study population was found to be considerably longer compared to the average time among additional bicyclists, suggesting that the obtained amount of bicycling may be obtainable. Our study does not present estimated yearly health impacts during the build-up time until all effects have been realised. Yearly health impact estimates due to the mode shift towards bicycling is for a steady-state situation. The literature on lag times between long-term air pollution exposure and morbidity/mortality is very limited. For physical activity these lag times are disease specific. The results are generalisable to settings with similar air pollution concentrations and injury risks.

## Conclusion

Using registry data of individuals’ home and work addresses and air pollution exposure calculations using a network of bicycle paths and roads available for bicycling, the largest health benefit was estimated due to the increased amount of physical activity. However, considerable health gains were also estimated in the population due to decreased air pollution exposure. The net health benefit is considerable also after considering the risk of traffic injuries based on national hospital injury data.
